# Tilted Fiber Bragg Grating Sensor Using Chemical Plating of a Palladium Membrane for the Detection of Hydrogen Leakage

**DOI:** 10.3390/s18124478

**Published:** 2018-12-18

**Authors:** Jiachen Yu, Zhenlin Wu, Xin Yang, Xiuyou Han, Mingshan Zhao

**Affiliations:** 1 School of Optoelectronic Engineering and Instrumentation Science, Dalian University of Technology, Dalian 116023, China; yu_jiachen@mail.dlut.edu.cn (J.Y.); xyhan@dlut.edu.cn (X.H.); mszhao@dlut.edu.cn (M.Z.); 2 Department of Electrical and Electronics Engineering, School of Engineering, Cardiff University, Cardiff CF10 3AT, UK; yangx26@cardiff.ac.uk

**Keywords:** optical fiber hydrogen sensor, palladium membrane, electroless plating, tilted fiber Bragg grating

## Abstract

A tilted fiber Bragg grating (TFBG) hydrogen sensor coated with a palladium (Pd) membrane by the electroless plating method is proposed in this paper. A uniform layer of Pd metal is fabricated in aqueous solutions by the chemical coating method, which is used as the sensitive element to detect the change of the surrounding refractive index (SRI) caused by hydrogen absorption. The change in SRI causes an unsynchronized change of the cladding modes and the Bragg peak in the TFBG transmission spectrum, thereby eliminating the cross-sensitivity due to membrane expansion and is able to simultaneously monitor the presence of cracks in the pipe, as well as the hydrogen leakage. By subtracting the wavelength shift caused by fiber expansion, the change of SRI, i.e., the information from the H_2_ level, can be separately obtained. The drifted wavelength is measured for the H_2_ concentration below the hydrogen explosion limit between 1% and 4%. The chemical-based coating has the advantages of a low cost, a simple operation, and being suitable for coating on long fiber structures. The proposed sensor is able to detect the H_2_ signal in 5 min at a 1% H_2_ concentration. The proposed sensor is proved to be able to monitor the hydrogen level without the cross-sensitivity of temperature variation and expansion strains, so could be a good candidate for security applications in industry.

## 1. Introduction

Hydrogen is considered an ideal clean energy and efficient fuel. At present, liquid hydrogen has been widely used in the aerospace field. Hydrogen-oxygen fuel cells can also be used to convert driving energy from hydrogen for automobiles. However, due to the unique nature of hydrogen, its storage and pipeline transportation is very difficult and it can be dangerous once it has leaked. At room temperature and standard atmospheric pressure, the explosive concentration of hydrogen can be as low as 4% in volume percentage [1]. Therefore, a safe and effective method for detecting hydrogen is urgently needed. Compared with traditional electronic sensors, optical sensors have many advantages, including electrical passiveness and insensitivity to electromagnetic interference [2]. Moreover, due to the flammable and explosive nature of hydrogen, the use of optical fiber detection can be much safer. Currently, various types of hydrogen sensors based on optical fibers have been proposed, including fiber Bragg gratings (FBG) [3,4], specially treated fibers (side polishing fiber [5] and singlemode-multimode-singlemode fiber [6]), and micro-cantilever beam fiber Bragg grating [7], as well as TFBG sensors using the SPR effect [8]. Most of the sensors use a palladium (Pd) metal membrane as the hydrogen sensing element, due to its strong ability to absorb hydrogen [7]. Once the Pd film absorbs hydrogen, the metal lattice experiences an α–β phase transition, which can be detected by various sensing structures. However, these types of hydrogen fiber sensors require special fibers or transform the sensing element into special forms in order to improve the sensitivity, which will introduce great difficulty to the production of the sensors. Taking the FBG hydrogen sensor as an example, Fisser et al. [9] used a layer of Pd membrane with a certain morphology to wrap the FBG, and the FBG spectrum could be changed by the expansion of Pd after hydrogen absorption. In practice, many other factors can also affect the FBG spectrum, such as temperature and changing stress caused by pipe cracking. Therefore, it can be helpful to develop a type of sensor which can detect the tube cracking and the hydrogen leakage at the same time, allowing the real-time monitoring of H_2_ leakage and pipe safety.

Tilted fiber Bragg grating (TFBG) is another type of fiber grating which has been investigated to solve this problem. TFBG is fabricated with the grating plane tilted at a certain angle with respect to the light propagation direction. Compared with FBG, except for a portion of the forward-directed light, which is coupled into the backward-directed core mode, the coupling of the core guided mode to the cladding mode and radiation mode also becomes stronger as the tilted angle increases, resulting in unique spectral characteristics [10]. Therefore, for sensing applications, the FBG sensor may not be able to distinguish the spectral changes caused by the surrounding environment or stress deformation. After the hydrogen absorption by the Pd layer, both the change of the refractive index and the expansion of the film can affect the output spectrum of FBG, which would cause inaccuracy of the sensing results. As TFBG is extremely sensitive to the change of refractive index of the environment, it can be utilized to detect the hydrogen concentration by measuring the change in the refractive index of the Pd membrane. At the same time, the deformation of the grating region due to other factors can also be detected.

For the preparation of the thin layer of metal coating, the magnetron sputtering method is commonly used [3,4,8,11,12,13,14]. A uniform layer of metal can be formed on the surface of the short fiber by rotating it during the sputtering. However, for long fibers, such as TFBG or FBG, this method can be difficult to apply. In order to simplify the coating process with reduced costs and manufacturing difficulties, a new method of electroless coating is utilized. During the chemical reaction, Pd particles are generated and slowly condense on the surface of the fiber, forming a uniform thin layer of Pd over the grating area [6,15,16,17,18].

In this paper, an optical sensor based on TFBG is proposed which uses a chemically coated Pd layer as the sensing material. Through the pretreatment “sensitization and activation” and chemical coating, a dense Pd membrane will grow on the surface of the fiber. Through experimental results combined with calculations, the proposed TFBG sensor can accurately monitor pipe cracks and hydrogen leakage at the same time at different temperatures.

## 2. Sensing Principle

**Figure 1:**
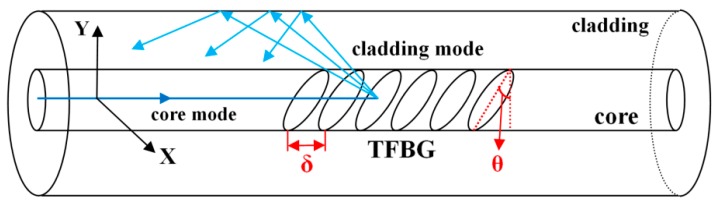
A schematic of TFBG with a tilt angle *θ* inscribed in the core of SMF.

**Figure 2:**
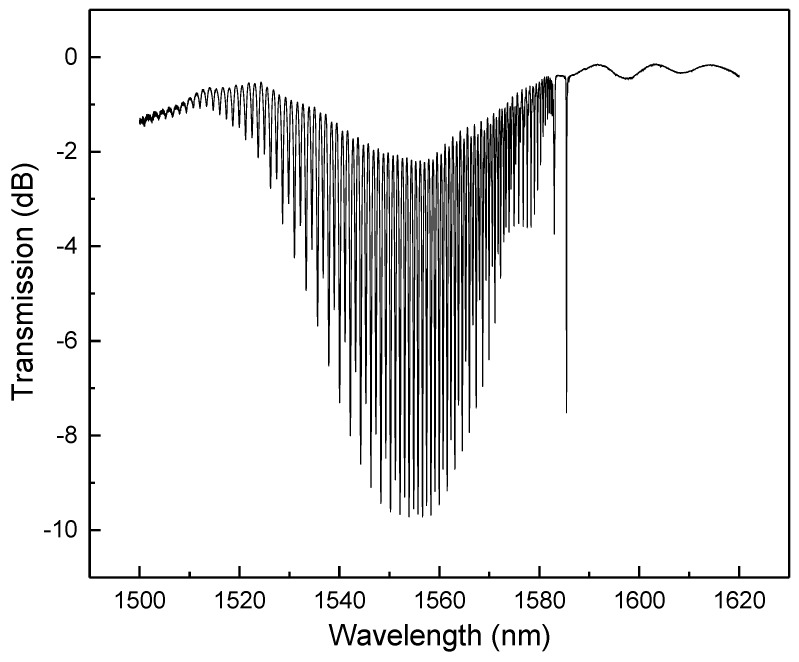
Transmission spectrum of 8° TFBG used in this paper.

A schematic diagram of TFBG with a tilt angle *θ* is shown in Figure 1. The grating structure is inscribed in the core of a single mode fiber (SMF). When the optical signal propagating in the fiber core reaches the grating region, a part of the signal will be reflected by the TFBG, forming a backward-propagating cladding mode. Similar to FBG, the transmission spectrum of TFBG also has a Bragg peak. Figure 2 shows the transmission spectrum of an 8° TFBG with a series of cladding modes and a Bragg peak.

The 8° angle TFBG allows the transmission spectrum to have sufficiently clear Bragg peak and cladding modes. The higher order of the cladding mode corresponds to the shorter wavelength and larger peak shifts for the change of external environment. However, if the order of the cladding mode is too high, the peak can be unstable, which is not conducive to quantitative measurement.

The wavelength of the Bragg peak and the *i*-th cladding mode of the TFBG spectrum can be expressed as [19]

(1) $$\lambda_{Bragg}=2\times n_{core,eff}\times \frac{\delta }{\cos\theta }$$

(2) $$\lambda_{clad}^{i}=\frac{\left(n_{core,eff}+n_{clad,eff}^{i}\right)\times \delta }{\cos\theta }$$

where *δ* is the spacing period of the grating; *θ* is the tilt angle of the grating; and $n_{core,eff}$ and $n_{clad,eff}^{i}$ represent the effective refractive index of the core and *i*-th order of cladding mode, respectively.

As the cladding modes transmit in the fiber, their effective refractive index can be affected by the surrounding refractive index (SRI), leading to a shift of the wavelength of cladding mode peaks. As the effective refractive index of the fiber core and the period of TFBG will not change with the SRI, i.e., $\frac{\partial n_{core,eff}}{\partial n_{SRI}}=\frac{\partial \delta }{\partial n_{SRI}}=0$. The relationship between the change of SRI and wavelength shift can be simplified as Equation (3).

(3) $$\Delta \lambda_{clad}^{i}=\frac{\delta }{\cos\theta }\cdot \frac{n_{clad,eff}^{i}}{\partial n_{SRI}}\cdot \Delta n_{SRI}$$

where $\Delta n_{SRI}$ is the change of SRI.

When the Pd film absorbs hydrogen, the lattices’ transition from *α* phase to *β* phase will cause changes to the refractive index, resulting in a variation of the SRI of the TFBG. The higher the hydrogen concentration is, the faster and larger the SRI of the Pd film changes, which leads to a wavelength drift of the TFBG transmission spectrum.

## 3. Sensor Fabrication

### 3.1. Sensitization and Activation (S&A)

In this experiment, the SMF-28 corning telecom fiber was loaded in a high pressure hydrogen environment for two weeks. Then TFBGs were inscribed using a 248 nm UV excimer laser and the phase-mask method [20]. The laser pulse was set to 6 mJ/150 Hz and a 15 mm long TFBG was made by a scanning technique. By adjusting the angle and the period of the phase-mask, a TFBG with an 8° tilted angle at a 1550 nm working wavelength was prepared.

After that, the grating section of the fiber was sonicated in acetone, anhydrous ethanol, and deionized (DI) water for 10 min, respectively. For the cleaning process, the acetone and DI water were used to dissolve the fat-soluble and water-soluble dirt, respectively. Anhydrous ethanol was used to clean up the acetone residue left on the fiber surface. The fiber was then dried in the oven at 60 °C for 20 min.

The sensitization and activation (S&A) process was employed to grow Pd particles on the fiber surface. This process requires repeatedly dripping reactive solutions onto the surface of the fiber, which are at low concentrations to maintain the reaction occurring at a very low rate. The process relies on the low concentration, as well as the small amount, of two reactive solutions to make sure that the slowly formed Pd particles can condense onto the surface of the fiber. By repeating this process, eventually, a large amount of Pd nuclei will be formed. These condensation nuclei can facilitate the process of chemical coating. The reaction rate needs to be limited. A faster reaction rate of electroless plating will form larger flocculated Pd particles and form precipitates.

The sensitization solution was prepared using 0.30 g SnCl_2_·2H_2_O dissolved in 50 mL 36% hydrochloric acid. Activation solution was prepared using 0.02 g PdCl_2_ dissolved in 50 mL 36% hydrochloric acid. The above chemical reagents were purchased from Liaodong Chemical Reagent Factory of Dalian (China), with the purity of analytical reagent (AR). By using the same method, the TFBG section of the fiber was immersed in two solutions for about 20 s each time, several times. The initial S&A step was completed until sparse Pd particles were homogeneously formed on the fiber surface. The chemical reaction that occurred during the process can be expressed as Equation (4):

(4) $${\mathrm{Sn}}^{2+}+{\mathrm{Pd}}^{2+}={\mathrm{Sn}}^{4+}+\mathrm{Pd}↓$$

**Figure 3:**
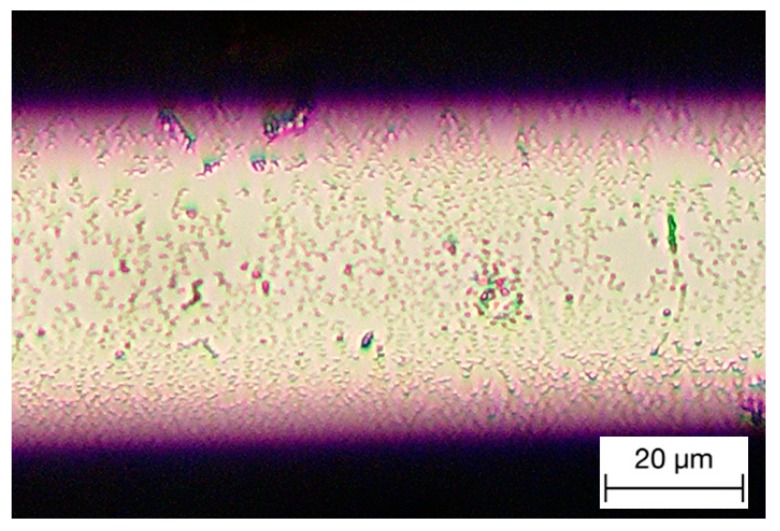
TFBG processed through drop and dip processes in microscope.

A new sensitization and activation method has been developed in this work to densify the crystallized Pd particles on the fiber, which is referred to as the “drop and dip” (D&D) method. Here, two pipettes are used to mix the solution on the top of the fiber surface. In this way, the reaction can be localized within the solution drops, and therefore, more Pd particles can condense onto the grating area. After several D&D processes, dense Pd particles can be formed on the fiber surface, as shown in Figure 3.

### 3.2. Chemical Coating Process

A dense layer of Pd coating is prepared during this process in a heated water bath environment. The water bath temperature affects both the reaction speed and the formation of the Pd film around the fiber. It is found that a higher temperature can facilitate the reaction speed. If the reaction speed is too slow, the Pd particles cannot form a homogeneous film; however, if the speed is too fast, the Pd particles tend to precipitate too fast and subside in the solution before being coated onto the fiber surface.

The chemical reaction requires 1 g/L PdCl_2_, 50 g/L Na_2_EDTA·2H_2_O, 260 mL/L 25% ammonia, 10 mL/L 36% hydrochloric, and 0.04 mol/L N_2_H_4_·H_2_O. By growing on top of the nucleus condensed in the S&A process, a uniform layer of Pd film can be prepared. The reaction depends on hydrazine to reduce Pd^2+^, which needs to be performed under an alkaline condition. If Pd particles form too fast, they will agglomerate with each other to form precipitates that will not grow on the fiber. The role of Na_2_EDTA is to form coordination compounds with Pd^2+^, which can reduce the reaction rate, and the same is true for ammonia. The Cl^−^ of hydrochloric can increase the solubility of Pd^2+^. Because the amount of hydrochloric acid is small, it will not affect the alkaline environment of the solution. The chemical reaction that occurred during the process can be expressed as Equation (5):

(5) $$2{\mathrm{Pd}}^{2+}+N_{2}H_{4}+4{\mathrm{OH}}^{-}\to 2\mathrm{Pd}↓+N_{2}↑+4H_{2}O$$

For the chemical coating process, Na_2_EDTA·2H_2_O and ammonia were dissolved in 300 mL DI water and heated to 60 °C to form the alkaline solution. The PdCl_2_ was then dissolved in hydrochloric and added to the above solution. Since the amount of hydrochloric is very small compared with the amount of ammonia, it will not affect the alkaline environment of the solution. After Pd^2+^ was completely dispersed at 60 °C, N_2_H_4_·H_2_O was slowly added to start the reaction and formed a layer of Pd on top of the fiber surface.

**Figure 4:**
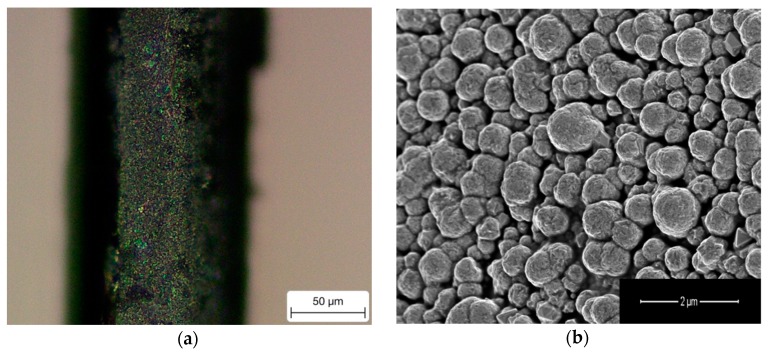
Microscopic images of coated Pd layer on the Pd-plated fiber surface. (**a**) Optical microscope of the fiber surface. (**b**) SEM imagine showing the uniform Pd particles condensed on the fiber, forming the Pd layer.

In this experiment, an optimal water bath temperature of 60 °C was determined to obtain a high quality of Pd coating, as shown in Figure 4. It can be found that a layer of homogenous Pd coating is formed on the fiber surface with the particle size around 1 μm, see Figure 4b. The scanning electronic microscopic (SEM) image was taken using Nova NanoSEM 450 field emission scanning electron microscopy (FEI Company, Hillsboro, OR, USA).

## 4. Results and Discussion

**Figure 5:**
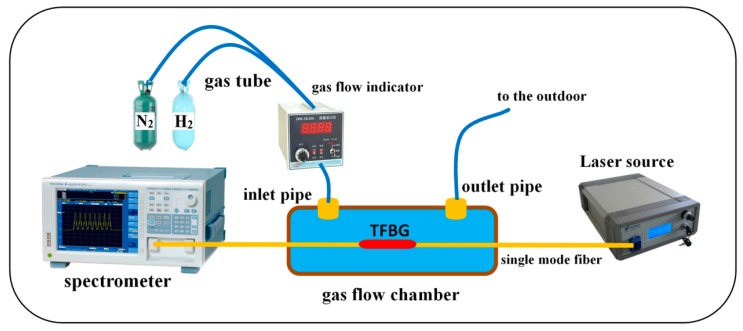
The schematic of gas flow chamber.

Figure 5 shows the schematic of the entire experimental setup. The Pd-coated TFBG is placed in a gas flow chamber, which is fastened onto the optical tablet to avoid vibration. The two ends of the gas flow chamber are sealed by glue to ensure gas tightness. The mixed gas enters and exits through the inlet pipe and the outlet pipe, respectively.

A broadband source (BBS) working between 1500 nm and 1620 nm with the power of 17 dBm (at 25 °C) was used in this experiment. The output spectrum was monitored using an optical spectrum analyzer (OSA) (AQ6370 model, Yokogawa, Japan) with a minimum wavelength resolution of 0.05 nm.

**Figure 6:**
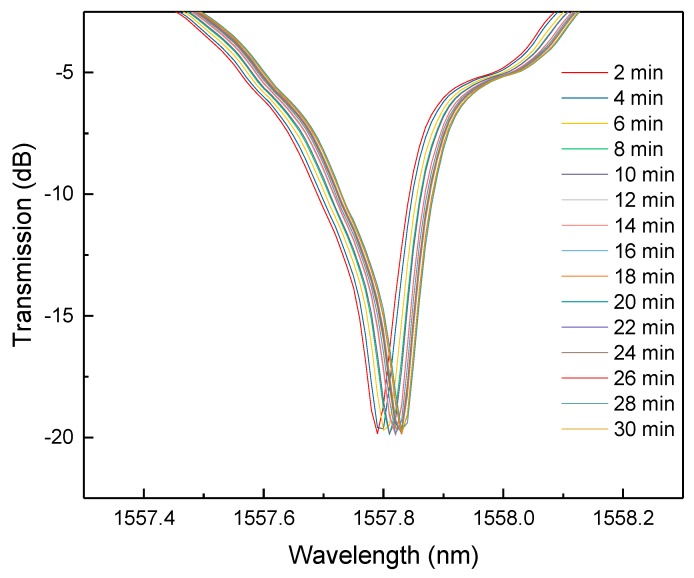
One of the peaks of cladding mode changes with hydrogen absorption.

The experiment was performed several times at different hydrogen concentrations. The Pd film absorbs hydrogen continuously during the testing until it reaches an equilibrium state. During this process, the Pd film expands, causing the change of SRI of the grating and the peak shift of the cladding mode. Figure 6 shows the continuous peak shift of a certain cladding mode at 4% hydrogen atmosphere over 30 min.

From Equations (1)–(3), it can be found that two main factors can cause the peak drift of the output spectrum, which are the refractive index change of the Pd film due to hydrogen absorption and the grating deformation caused by the Pd film expansion. In the experiment, the measurements were carried out under constant temperature conditions, so there is no effect of temperature on the amount of spectral drift. The grating deformation due to metal film expansion affects both the Bragg peak (Δ*B*) and the cladding mode (Δ*C*), leading to the same wavelength shift. However, the cladding modes can only be affected by the change of the refractive index of the metal film around the grating area. The difference of these two drifts (Δ*C*-Δ*B*) only gives the information of the hydrogen-related changes. For Δ*C*-Δ*B* = 0, it suggests no change of SRI, meaning no hydrogen leakage can be detected. In this case, if Δ*B* ≠ 0, it means a small crack may occur at the pipe, but has not caused a leakage yet. However, the case of Δ*C*-Δ*B* ≠ 0 indicates that leaked hydrogen can be detected in the atmosphere. By measuring the Δ*B* and Δ*C* at the same time, both the pipeline cracks and the hydrogen leakage can be monitored at the same time.

**Figure 7:**
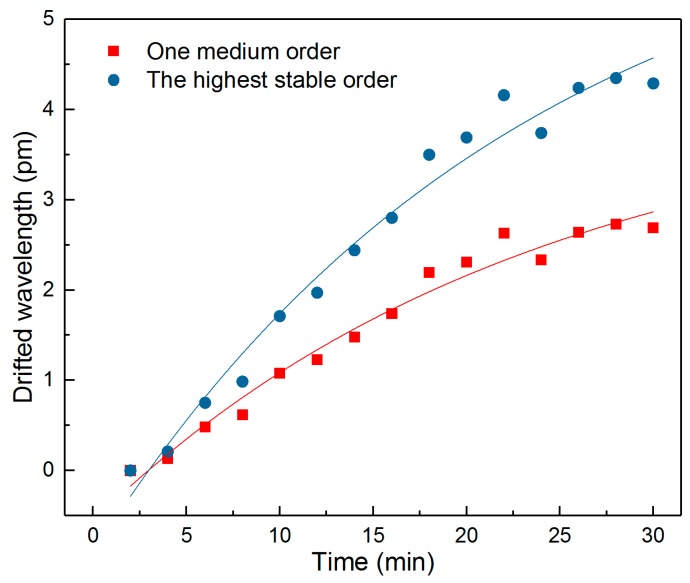
The fitting curves of Δ*C*-Δ*B* for different orders of cladding modes at same H_2_ concentration.

For the wavelength shift of the output spectrum, it can be found that different orders of the cladding mode have different Δ*C*-Δ*B*. A higher order of the cladding mode has a larger wavelength drift. Figure 7 shows the drifted wavelength (Δ*C*-Δ*B*) for the highest stable order of cladding mode (at 1526.20 nm) and one of a medium order of cladding mode (at 1559.98 nm) at a 2% hydrogen concentration. A roughly 80% increase of the wavelength drift can be detected in the highest order curve compared with the medium order one at 30 min, which gives high sensitivity during the measurement. Therefore, in this experiment, the wavelength change of the highest order cladding mode is used for sensing purposes.

**Figure 8:**
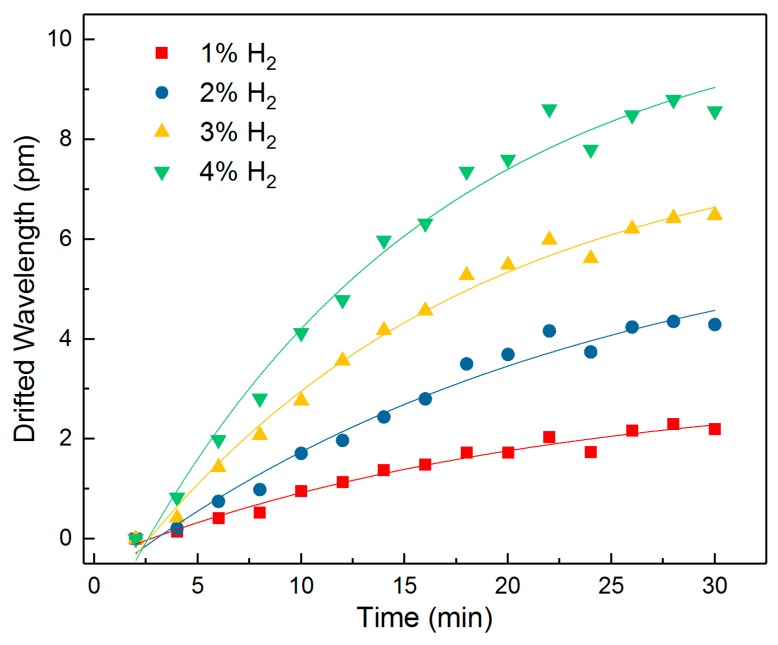
The wavelength drift (Δ*C*-Δ*B*) at different H_2_ concentrations with the fitting curves.

The same TFBG sensor was tested at the same temperature under 1%, 2%, 3%, and 4% hydrogen concentration conditions, respectively. Figure 8 shows the peak drift fit curve of Δ*C*-Δ*B* over time. The sensor was placed in a ventilated environment for a sufficient time to allow the hydrogen to overflow. It can be seen that the higher the hydrogen concentration, the larger and faster the drift (Δ*C*-Δ*B*) varies. The value of Δ*C*-Δ*B* gradually rises as the time increases and tends to stabilize after approximately 20 min.

**Figure 9:**
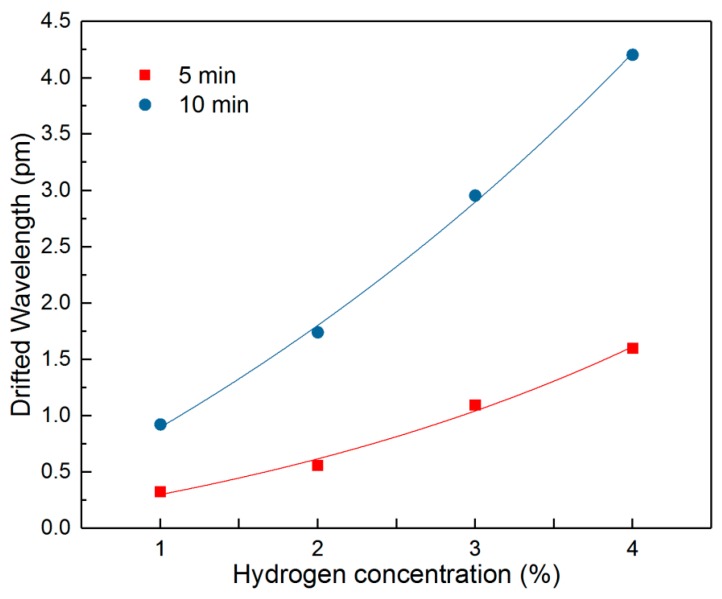
The fitting curve of Δ*C*-Δ*B* in different hydrogen concentrations for 5 min and 10 min.

The relationships between the wavelength drift and the hydrogen concentration for 5 min and 10 min are plotted in Figure 9. Both curves can be fitted as follows:

(6) $$y=0.695\times e^{\frac{x}{3.410}}-0.631$$

(7) $$y=3.596\times e^{\frac{x}{5.281}}-3.448$$

It can be seen that the peak drift of Δ*C*-Δ*B* for both curves shows an exponentially increasing trend as the hydrogen concentration grows. The 5 min curve already shows a clear trend with increasing H_2_ concentration, indicating a short sensing time for the proposed sensor. For the low H_2_ concentration of 1%, the wavelength drift rises to 1 pm after 10 min, which shows a strong ability for low concentration sensing.

For a high concentration and exposure time, the pure Pd layer may have a hydrogen brittle effect, leading to the fast transition from alpha phase to beta phase and forming small cracks on the membrane surface, which can affect the sensitivity of the sensor. One way to overcome this effect is to dope gold or silver into the film and form alloys of Pd/Au and Pd/Ag. Further improvement of the sensor will include an investigation of the co-deposition of the alloy metal layer using the proposed electroless chemical plating method in this paper for better sensor sensitivity and reliability.

Compared to the traditional H_2_ sensors, the sensor proposed in this work has the advantages of electrical passiveness, a lower cost, and being easy to fabricate. In addition, it can monitor the cracking of an H_2_ pipeline and detect hydrogen leakage at the same time, which makes it a good candidate for monitoring the pipeline in the industry.

## 5. Conclusions

In this paper, a new method for reducing Pd^2+^ in aqueous solution for plating Pd film on optical fibers is proposed, which can form a dense and uniform film on a long fiber surface easily and inexpensively. By depositing a Pd membrane on the surface of an 8° tilted TFBG, its spectrum can have a specific response to different hydrogen concentrations with a high sensitivity. The sensor can monitor the crack of a pipeline while only responding to hydrogen by calculation. For a 4% hydrogen concentration, the calculated wavelength can drift by 1 pm in less than 5 min. For the same situation of 1%, the time will be in 10 min. The spectral drift of non-hydrogen-specific detection without calculation will be much larger. The result of this study provides a new method for coating Pd films on optical fibers, and will be useful for hydrogen sensing of TFBG in the near future.
